# (Pyridine-2-aldoximato-κ^2^ *N*,*N*′)bis­[2-(pyridin-2-yl)phenyl-κ^2^ *C* ^1^,*N*]iridium(III)

**DOI:** 10.1107/S1600536813004297

**Published:** 2013-02-20

**Authors:** Satyanarayan Pal, Bimal Chandra Singh

**Affiliations:** a Department of Chemistry, International Institute of Information Technology, Bhubaneswar, Odisha 751 003, India

## Abstract

In the title complex, [Ir(C_11_H_8_N)_2_(C_6_H_5_N_2_O)], the octa­hedrally coordinated Ir^III^ atom is bonded to two 2-(pyridin-2-yl)phenyl ligands, through two phenyl C and two pydidine N atoms, and to one pyridine-2-aldoxime ligand through a pyridine N and an oxime N atom. The oxime O atom of the aldoxime unit forms inter­molecular C—H⋯O hydrogen bonds, which result in a two-dimensional hydrogen-bonded polymeric network parallel to (100). C—H⋯π inter­actions are also observed.

## Related literature

For the synthesis of the iridium phenyl­pyridine starting material, see: Nonoyama (1974 ▶). For preparation of phenyl pyridine-based Ir(III) complexes, see: Lamansky *et al.* (2001 ▶). For similar types of complexes, see: Neve *et al.* (1999 ▶). For standard bond lengths, see: Allen *et al.* (1987 ▶). For hydrogen bonding, see: Desiraju (1991 ▶) and for C—H⋯π inter­actions, see: Ma & Dougherty (1997 ▶). For oxime ligands, see: Godycki & Rundle (1953 ▶).
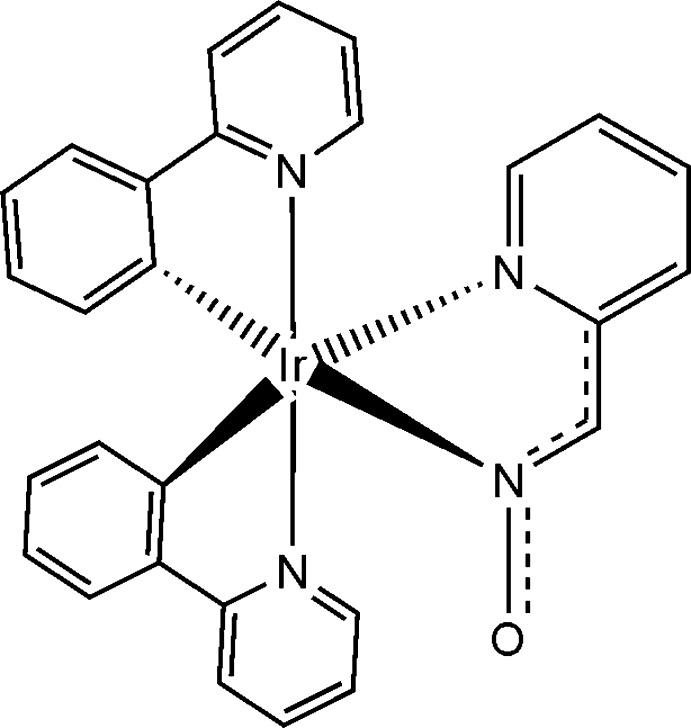


## Experimental

### 

#### Crystal data

[Ir(C_11_H_8_N)_2_(C_6_H_5_N_2_O)]
*M* *_r_* = 621.71
Monoclinic, 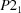

*a* = 9.414 (1) Å
*b* = 14.226 (2) Å
*c* = 9.551 (1) Å
β = 117.260 (7)°
*V* = 1137.0 (2) Å^3^
*Z* = 2
Mo *K*α radiation
μ = 5.90 mm^−1^
*T* = 293 K
0.40 × 0.32 × 0.24 mm

#### Data collection

Bruker SMART CCD area-detector diffractometer
Absorption correction: multi-scan (*SADABS*; Bruker, 2003 ▶) *T*_min_ = 0.201, *T*_max_ = 0.332
25091 measured reflections
6898 independent reflections
5718 reflections with *I* > 2σ(*I*)
*R*_int_ = 0.065

#### Refinement

*R*[*F* ^2^ > 2σ(*F* ^2^)] = 0.037
*wR*(*F* ^2^) = 0.063
*S* = 0.96
6898 reflections
308 parameters
1 restraint
H-atom parameters constrained
Δρ_max_ = 1.34 e Å^−3^
Δρ_min_ = −0.83 e Å^−3^
Absolute structure: Flack (1983 ▶), 3193 Friedel pairs
Flack parameter: 0.007 (10)

### 

Data collection: *SMART* (Bruker, 2003 ▶); cell refinement: *SMART* (Bruker, 2003 ▶); data reduction: *SAINT* (Bruker, 2003 ▶); program(s) used to solve structure: *SHELXS97* (Sheldrick, 2008 ▶); program(s) used to refine structure: *SHELXL97* (Sheldrick, 2008 ▶); molecular graphics: *ORTEP-3 for Windows* (Farrugia, 2012 ▶); software used to prepare material for publication: *SHELXL97*.

## Supplementary Material

Crystal structure: contains datablock(s) I, global. DOI: 10.1107/S1600536813004297/bg2497sup1.cif

Structure factors: contains datablock(s) I. DOI: 10.1107/S1600536813004297/bg2497Isup2.hkl

Additional supplementary materials:  crystallographic information; 3D view; checkCIF report

## Figures and Tables

**Table 1:** Hydrogen-bond geometry (Å, °) *Cg*1, *Cg*2 and *Cg*3 are the centroids of the C17–C22, N2/C12–C16 and N1/C1–C5 rings, respectively.

*D*—H⋯*A*	*D*—H	H⋯*A*	*D*⋯*A*	*D*—H⋯*A*
C7—H7⋯O1^i^	0.93	2.35	3.235 (10)	159
C15—H15⋯O1^ii^	0.93	2.37	3.239 (9)	155
C3—H3⋯*Cg*1^iii^	0.93	2.74	3.604 (7)	155
C8—H8⋯*Cg*2^i^	0.93	2.69	3.606 (7)	170
C13—H13⋯*Cg*3^iv^	0.93	2.71	3.538 (9)	148

Symmetry codes: (i) 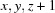; (ii) 
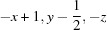
; (iii) 
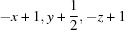
; (iv) 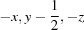.

**Fig. 1.**
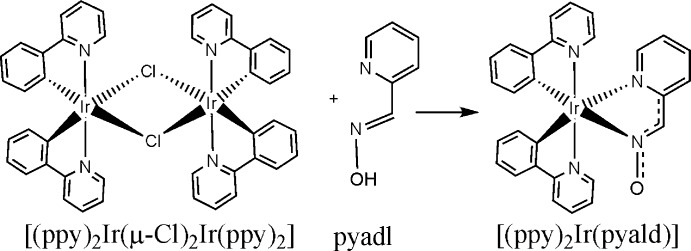
Synthetic scheme of the title complex showing the starting material and ligand.

**Fig. 2.**
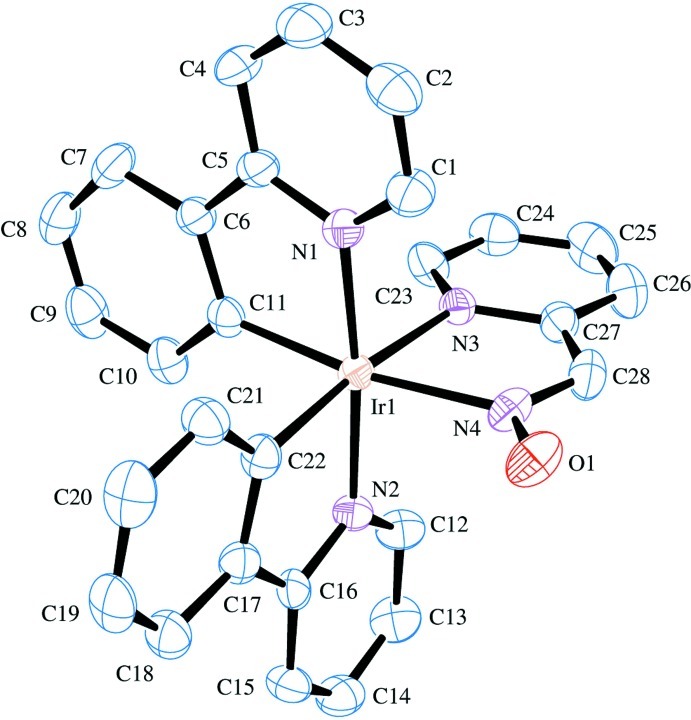
Molecular structure of the title complex with the atom numbering scheme. The displacement ellipsoids are shown at 50% probability level. Hydrogen atoms are omitted for clarity.

**Fig. 3.**
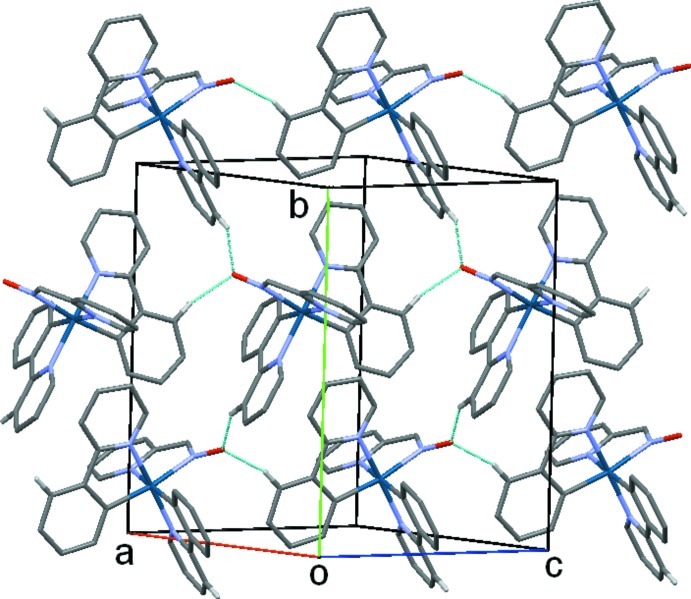
A view of the hydrogen bonded (100) two dimensional polymeric network. Cyan coloured lines indicate the H-bond.

## supplementary crystallographic information

### Comment

Herein we describe the crystal structure of a neutral Ir(III) octahedral complex, namely [Ir(ppy)_2_(pyald)] (ppy = 2-phenylpyridine, pyald=2-pyridinealdoxime). The asymmetric unit is shown in Figure 2, which depicts that the Ir centre is coordinated by two 2-phenyl pyridine and one 2-pyridinealdoxime ligands. Two pyridine-N atoms from ppy moieties occupy the axial positions, whereas two phenyl-C from ppy ligands and two N atoms from the 2-pyridinealdoxime unit form the square plane. All three ligand tether the metal through five membered chelate rings, which ultimately lead to a distorted octahedral coordination geometry. The *trans* N1—Ir1—N2, C11—Ir1—N4 and C22—Ir1—N3 angles are 173.75 (19)°, 170.6 (2)° and 173.4 (18)° respectively ( ideal value: 180°). Corresponding *cis* angles show similar small deviation from 90°, for example, *cis* C22—Ir1—C11, C11—Ir1—N1, N4—Ir1—N3, and C11—Ir1—N3 angles are 88.4 (2)°, 80.4 (2)°, 77.15 (18)° and 95.41 (19)° respectively. The two phenylpyridine ligands are nearly perpendicular as indicated by the dihedral angle between their least square planes, 87.60 (11)°. The third ligand 2-pyridinealdoxime has a similar nearly perpendicular orientation with the adjacent 2-phenylpyridines. The dihedral angles of 2-pyridineadloxime to 2-phenylpyridines are 85.09 (11)° and 89.54 (15)°. The mean Ir—C [2.019 (4) Å] and Ir—N [2.087 (2) Å] bond distances are consistent with values reported in literature (see, Neve *et al.*, 1999; Lamansky *et al.*, 2001). The oxime-O of 2-pyridinealdoxime is deprotonated and remain non-coordinated. This is an expected fact for oxime ligands where the oxime-N binds a metal centre in presence of a deprotonated oxime-O (see, Godycki *et al.*, 1953). The negative charge on the oxime-O is delocalized over the aldoxime moiety (Scheme1). This is supported by the N4—O1 [1.289 (6) Å] and C27—C28 [1.420 (8) Å] bond lengths, which clearly indicate their partial double bond character (see, Allen *et al.*, 1987). Thus the negative charge is distributed over the pyridinealdoxime moiety through O1, N4, C28 and the pyridine ring.

The packing scheme is ruled by C—H···O hydrogen bonds (see, Desiraju,1991), which ultimately lead to the formation of a two dimensional hydrogen bonded polymeric structure (Figure 3) and weaker intermolecular C—H···π interactions (see, Ma *et al.*, 1997). These interactions are presented in Table 1.

### Experimental

The iridium starting material [(ppy)_2_Ir(µ –Cl)_2_Ir(ppy)_2,_ ppy = 2-phenylpyridine] was prepared by following the procedure reported in the literature (see, Nonoyama,1974). The synthetic scheme of the title complex is shown in Figure 1. The detailed synthetic procedure is as follows:

In a 100 ml round bottom flask, 2-pyridinealdoxime (266 mg, 2.18 mmol) was taken in 25 ml of 2-methoxy ethanol and added with triehtyl amine (220.6 mg, 2.18 mmol). The mixture was thoroughly mixed by stirring and added with [(ppy)_2_Ir(µ –Cl)_2_Ir(ppy)_2_ ] (585 mg, 0.545 mmol) starting material. The resulted mixture was refluxed under a N_2_ atmosphere for 18 hrs. The yellow precipitate thus formed was filtered and dried under vacuum. The complex was purified on a neutral aluminium oxide column by eluting with dichloromethane. The first yellow band was discarded as starting material and the second yellow band was collected as the title compound. The solution was evaporated and the remaining solid washed with hexane and dried under vacuum to yield an yellow powder (140 mg, 41%).

### Refinement

All the non-H atoms were refined anisotropically. The ligands H atoms were included at idealized position using riding model with C—H = 0.93 Å and *U*_iso_(H) = 1.2Ueq

### Crystal data

**Table:**

[Ir(C_11_H_8_N)_2_(C_6_H_5_N_2_O)]	*F*(000) = 604
*M**_r_* = 621.71	*D*_x_ = 1.816 Mg m^−^^3^
Monoclinic, *P*2_1_	Mo *K*α radiation, λ = 0.71073 Å
Hall symbol: P 2yb	Cell parameters from 4353 reflections
*a* = 9.414 (1) Å	θ = 2.4–22.5°
*b* = 14.226 (2) Å	µ = 5.90 mm^−^^1^
*c* = 9.551 (1) Å	*T* = 293 K
β = 117.260 (7)°	Rod, yellow
*V* = 1137.0 (2) Å^3^	0.40 × 0.32 × 0.24 mm
*Z* = 2	

### Data collection

**Table:**

Bruker SMART CCD area-detector diffractometer	6898 independent reflections
Radiation source: fine-focus sealed tube	5718 reflections with *I* > 2σ(*I*)
Graphite monochromator	*R*_int_ = 0.065
φ and ω scans	θ_max_ = 30.6°, θ_min_ = 2.4°
Absorption correction: multi-scan (*SADABS*; Bruker, 2003)	*h* = −13→13
*T*_min_ = 0.201, *T*_max_ = 0.332	*k* = −20→19
25091 measured reflections	*l* = −13→13

### Refinement

**Table:**

Refinement on *F*^2^	Secondary atom site location: difference Fourier map
Least-squares matrix: full	Hydrogen site location: inferred from neighbouring sites
*R*[*F*^2^ > 2σ(*F*^2^)] = 0.037	H-atom parameters constrained
*wR*(*F*^2^) = 0.063	*w* = 1/[σ^2^(*F*_o_^2^) + (0.0158*P*)^2^] where *P* = (*F*_o_^2^ + 2*F*_c_^2^)/3
*S* = 0.96	(Δ/σ)_max_ < 0.001
6898 reflections	Δρ_max_ = 1.34 e Å^−^^3^
308 parameters	Δρ_min_ = −0.83 e Å^−^^3^
1 restraint	Absolute structure: Flack (1983), 3193 Friedel pairs
Primary atom site location: structure-invariant direct methods	Flack parameter: 0.007 (10)

### Special details

**Table:**

Geometry. All s.u.'s (except the s.u. in the dihedral angle between two l.s. planes) are estimated using the full covariance matrix. The cell s.u.'s are taken into account individually in the estimation of s.u.'s in distances, angles and torsion angles; correlations between s.u.'s in cell parameters are only used when they are defined by crystal symmetry. An approximate (isotropic) treatment of cell s.u.'s is used for estimating s.u.'s involving l.s. planes.
Refinement. Refinement of *F*^2^ against ALL reflections. The weighted *R*-factor *wR* and goodness of fit *S* are based on *F*^2^, conventional *R*-factors *R* are based on *F*, with *F* set to zero for negative *F*^2^. The threshold expression of *F*^2^ > 2σ(*F*^2^) is used only for calculating *R*-factors(gt) *etc*. and is not relevant to the choice of reflections for refinement. *R*-factors based on *F*^2^ are statistically about twice as large as those based on *F*, and *R*- factors based on ALL data will be even larger.

### Fractional atomic coordinates and isotropic or equivalent isotropic displacement parameters (Å^2^)

**Table:**

	*x*	*y*	*z*	*U*_iso_*/*U*_eq_	
Ir1	0.247960 (19)	0.64537 (3)	0.109712 (19)	0.02879 (5)	
C13	0.0866 (8)	0.3975 (4)	−0.1836 (8)	0.0513 (16)	
H13	−0.0105	0.3729	−0.2575	0.062*	
C11	0.2459 (6)	0.5892 (4)	0.3030 (7)	0.0342 (12)	
C15	0.3653 (8)	0.3892 (4)	−0.0391 (8)	0.0468 (16)	
H15	0.4604	0.3575	−0.0137	0.056*	
C20	0.7684 (7)	0.6230 (6)	0.3712 (8)	0.056 (3)	
H20	0.8552	0.6562	0.4458	0.068*	
C23	−0.1127 (5)	0.6426 (8)	0.0322 (6)	0.0425 (11)	
H23	−0.0789	0.6100	0.1264	0.051*	
N2	0.2292 (5)	0.5204 (3)	−0.0044 (5)	0.0326 (10)	
C17	0.5112 (7)	0.5240 (4)	0.1504 (7)	0.0371 (12)	
C5	0.2946 (7)	0.7521 (5)	0.3865 (7)	0.0352 (13)	
C27	−0.0502 (7)	0.7182 (4)	−0.1511 (7)	0.0371 (12)	
C1	0.3174 (8)	0.8495 (4)	0.2026 (8)	0.0448 (14)	
H1	0.3124	0.8572	0.1038	0.054*	
C12	0.0920 (8)	0.4817 (4)	−0.1096 (8)	0.0447 (14)	
H12	−0.0034	0.5127	−0.1337	0.054*	
N4	0.2216 (6)	0.7195 (3)	−0.0929 (6)	0.0380 (11)	
C24	−0.2732 (7)	0.6586 (7)	−0.0593 (8)	0.052 (2)	
H24	−0.3469	0.6387	−0.0260	0.062*	
C6	0.2659 (6)	0.6565 (8)	0.4195 (6)	0.0344 (19)	
C14	0.2258 (9)	0.3512 (4)	−0.1465 (8)	0.0499 (16)	
H14	0.2252	0.2941	−0.1943	0.060*	
N1	0.2887 (5)	0.7645 (3)	0.2439 (5)	0.0327 (10)	
C3	0.3588 (8)	0.9144 (4)	0.4456 (8)	0.0511 (16)	
H3	0.3837	0.9650	0.5145	0.061*	
C2	0.3544 (8)	0.9260 (4)	0.3018 (9)	0.0507 (16)	
H2	0.3758	0.9842	0.2714	0.061*	
C18	0.6659 (8)	0.4887 (5)	0.2082 (8)	0.0496 (16)	
H18	0.6829	0.4312	0.1721	0.060*	
C16	0.3700 (7)	0.4756 (4)	0.0350 (7)	0.0361 (12)	
C4	0.3268 (9)	0.8286 (4)	0.4882 (7)	0.0453 (15)	
H4	0.3264	0.8211	0.5848	0.054*	
O1	0.3361 (5)	0.7455 (3)	−0.1226 (5)	0.0514 (11)	
C28	0.0738 (8)	0.7441 (4)	−0.1892 (7)	0.0461 (14)	
H28	0.0509	0.7779	−0.2805	0.055*	
C10	0.2257 (8)	0.4959 (5)	0.3361 (8)	0.0429 (14)	
H10	0.2129	0.4497	0.2624	0.052*	
C9	0.2242 (9)	0.4701 (5)	0.4751 (9)	0.0549 (18)	
H9	0.2108	0.4073	0.4935	0.066*	
C25	−0.3231 (8)	0.7045 (5)	−0.2005 (9)	0.0605 (19)	
H25	−0.4313	0.7153	−0.2646	0.073*	
C22	0.4815 (6)	0.6111 (4)	0.2015 (6)	0.0359 (13)	
C26	−0.2123 (8)	0.7343 (5)	−0.2466 (8)	0.0549 (17)	
H26	−0.2456	0.7654	−0.3422	0.066*	
C19	0.7923 (7)	0.5379 (5)	0.3172 (8)	0.0536 (17)	
H19	0.8952	0.5139	0.3551	0.064*	
C8	0.2420 (8)	0.5359 (5)	0.5848 (7)	0.0529 (17)	
H8	0.2419	0.5178	0.6783	0.063*	
N3	−0.0031 (5)	0.6730 (3)	−0.0110 (5)	0.0325 (10)	
C7	0.2606 (7)	0.6308 (10)	0.5584 (7)	0.048 (2)	
H7	0.2692	0.6762	0.6320	0.058*	
C21	0.6150 (7)	0.6598 (6)	0.3147 (7)	0.043 (2)	
H21	0.6004	0.7174	0.3524	0.052*	

### Atomic displacement parameters (Å^2^)

**Table:**

	*U*^11^	*U*^22^	*U*^33^	*U*^12^	*U*^13^	*U*^23^
Ir1	0.02708 (8)	0.03172 (8)	0.02746 (9)	−0.0001 (2)	0.01240 (6)	−0.0010 (2)
C13	0.047 (4)	0.049 (4)	0.053 (4)	−0.014 (3)	0.019 (3)	−0.014 (3)
C11	0.024 (3)	0.040 (3)	0.036 (3)	0.004 (2)	0.012 (2)	0.004 (3)
C15	0.059 (4)	0.036 (3)	0.053 (4)	0.013 (3)	0.032 (4)	0.002 (3)
C20	0.033 (3)	0.076 (9)	0.053 (4)	−0.012 (3)	0.013 (3)	0.007 (4)
C23	0.035 (3)	0.043 (2)	0.055 (3)	0.005 (6)	0.025 (2)	0.012 (7)
N2	0.031 (3)	0.033 (2)	0.036 (3)	0.000 (2)	0.017 (2)	−0.002 (2)
C17	0.034 (3)	0.044 (3)	0.035 (3)	0.002 (2)	0.018 (3)	0.002 (2)
C5	0.023 (3)	0.048 (4)	0.033 (3)	0.006 (3)	0.011 (3)	−0.006 (3)
C27	0.037 (3)	0.035 (3)	0.032 (3)	0.000 (2)	0.009 (3)	−0.003 (2)
C1	0.052 (4)	0.038 (3)	0.043 (4)	−0.006 (3)	0.021 (3)	−0.003 (3)
C12	0.047 (4)	0.041 (3)	0.052 (4)	−0.007 (3)	0.028 (3)	−0.009 (3)
N4	0.047 (3)	0.039 (3)	0.031 (3)	−0.011 (2)	0.021 (3)	−0.006 (2)
C24	0.040 (3)	0.052 (6)	0.061 (4)	−0.002 (3)	0.022 (3)	−0.009 (4)
C6	0.029 (2)	0.039 (6)	0.034 (3)	0.003 (3)	0.013 (2)	−0.002 (3)
C14	0.068 (5)	0.035 (3)	0.054 (4)	−0.003 (3)	0.034 (4)	−0.007 (3)
N1	0.024 (2)	0.038 (2)	0.031 (2)	−0.0002 (19)	0.008 (2)	−0.0053 (19)
C3	0.050 (4)	0.044 (3)	0.049 (4)	−0.003 (3)	0.013 (3)	−0.017 (3)
C2	0.043 (4)	0.038 (3)	0.064 (5)	−0.002 (3)	0.017 (3)	−0.004 (3)
C18	0.040 (4)	0.064 (4)	0.048 (4)	0.013 (3)	0.023 (3)	0.011 (3)
C16	0.041 (3)	0.038 (3)	0.033 (3)	0.010 (2)	0.020 (3)	0.012 (2)
C4	0.052 (4)	0.047 (4)	0.032 (3)	0.001 (3)	0.015 (3)	−0.012 (3)
O1	0.055 (3)	0.062 (3)	0.048 (3)	−0.021 (2)	0.032 (2)	−0.006 (2)
C28	0.049 (4)	0.051 (4)	0.032 (3)	0.003 (3)	0.012 (3)	0.008 (3)
C10	0.035 (4)	0.046 (4)	0.048 (4)	0.003 (3)	0.019 (3)	0.010 (3)
C9	0.050 (4)	0.057 (4)	0.064 (5)	0.009 (3)	0.032 (4)	0.025 (4)
C25	0.030 (4)	0.063 (4)	0.068 (5)	0.003 (3)	0.006 (3)	−0.005 (4)
C22	0.028 (3)	0.045 (3)	0.033 (3)	0.000 (2)	0.013 (2)	0.003 (2)
C26	0.045 (4)	0.058 (4)	0.048 (4)	0.003 (3)	0.010 (3)	0.011 (3)
C19	0.024 (3)	0.082 (5)	0.051 (4)	0.010 (3)	0.014 (3)	0.015 (4)
C8	0.052 (4)	0.073 (5)	0.036 (4)	−0.001 (3)	0.022 (3)	0.009 (3)
N3	0.030 (2)	0.034 (3)	0.030 (2)	0.0030 (16)	0.0109 (19)	−0.0012 (16)
C7	0.043 (3)	0.071 (7)	0.030 (3)	0.001 (4)	0.018 (2)	−0.005 (4)
C21	0.038 (3)	0.041 (6)	0.046 (3)	0.002 (3)	0.015 (3)	−0.003 (3)

### Geometric parameters (Å, º)

**Table:**

Ir1—C22	2.018 (5)	C1—N1	1.337 (7)
Ir1—C11	2.020 (6)	C1—C2	1.379 (8)
Ir1—N2	2.051 (4)	C1—H1	0.9300
Ir1—N1	2.053 (4)	C12—H12	0.9300
Ir1—N4	2.116 (5)	N4—O1	1.289 (6)
Ir1—N3	2.139 (4)	N4—C28	1.316 (8)
C13—C14	1.362 (9)	C24—C25	1.374 (10)
C13—C12	1.379 (8)	C24—H24	0.9300
C13—H13	0.9300	C6—C7	1.399 (8)
C11—C10	1.397 (8)	C14—H14	0.9300
C11—C6	1.414 (10)	C3—C4	1.362 (9)
C15—C14	1.355 (9)	C3—C2	1.365 (9)
C15—C16	1.409 (8)	C3—H3	0.9300
C15—H15	0.9300	C2—H2	0.9300
C20—C19	1.374 (11)	C18—C19	1.362 (9)
C20—C21	1.392 (9)	C18—H18	0.9300
C20—H20	0.9300	C4—H4	0.9300
C23—N3	1.345 (7)	C28—H28	0.9300
C23—C24	1.374 (8)	C10—C9	1.384 (9)
C23—H23	0.9300	C10—H10	0.9300
N2—C12	1.339 (7)	C9—C8	1.357 (10)
N2—C16	1.360 (7)	C9—H9	0.9300
C17—C18	1.392 (8)	C25—C26	1.372 (10)
C17—C22	1.407 (7)	C25—H25	0.9300
C17—C16	1.454 (8)	C22—C21	1.408 (8)
C5—N1	1.348 (7)	C26—H26	0.9300
C5—C4	1.397 (8)	C19—H19	0.9300
C5—C6	1.449 (13)	C8—C7	1.399 (16)
C27—N3	1.363 (7)	C8—H8	0.9300
C27—C26	1.392 (9)	C7—H7	0.9300
C27—C28	1.420 (8)	C21—H21	0.9300
			
C22—Ir1—C11	88.4 (2)	C11—C6—C5	115.8 (5)
C22—Ir1—N2	80.4 (2)	C15—C14—C13	119.2 (6)
C11—Ir1—N2	96.2 (2)	C15—C14—H14	120.4
C22—Ir1—N1	94.21 (19)	C13—C14—H14	120.4
C11—Ir1—N1	80.4 (2)	C1—N1—C5	119.8 (5)
N2—Ir1—N1	173.75 (19)	C1—N1—Ir1	124.6 (4)
C22—Ir1—N4	99.6 (2)	C5—N1—Ir1	115.6 (4)
C11—Ir1—N4	170.6 (2)	C4—C3—C2	120.0 (6)
N2—Ir1—N4	90.0 (2)	C4—C3—H3	120.0
N1—Ir1—N4	93.99 (17)	C2—C3—H3	120.0
C22—Ir1—N3	173.47 (18)	C3—C2—C1	118.5 (6)
C11—Ir1—N3	95.41 (19)	C3—C2—H2	120.7
N2—Ir1—N3	93.90 (17)	C1—C2—H2	120.7
N1—Ir1—N3	91.65 (16)	C19—C18—C17	120.5 (6)
N4—Ir1—N3	77.15 (18)	C19—C18—H18	119.8
C14—C13—C12	118.8 (6)	C17—C18—H18	119.8
C14—C13—H13	120.6	N2—C16—C15	117.9 (6)
C12—C13—H13	120.6	N2—C16—C17	115.1 (5)
C10—C11—C6	116.6 (6)	C15—C16—C17	127.0 (5)
C10—C11—Ir1	129.9 (5)	C3—C4—C5	119.8 (6)
C6—C11—Ir1	113.4 (5)	C3—C4—H4	120.1
C14—C15—C16	121.7 (6)	C5—C4—H4	120.1
C14—C15—H15	119.2	N4—C28—C27	118.8 (5)
C16—C15—H15	119.2	N4—C28—H28	120.6
C19—C20—C21	120.4 (6)	C27—C28—H28	120.6
C19—C20—H20	119.8	C9—C10—C11	122.1 (7)
C21—C20—H20	119.8	C9—C10—H10	119.0
N3—C23—C24	121.8 (6)	C11—C10—H10	119.0
N3—C23—H23	119.1	C8—C9—C10	120.4 (6)
C24—C23—H23	119.1	C8—C9—H9	119.8
C12—N2—C16	119.9 (5)	C10—C9—H9	119.8
C12—N2—Ir1	125.0 (4)	C26—C25—C24	119.6 (6)
C16—N2—Ir1	115.1 (4)	C26—C25—H25	120.2
C18—C17—C22	121.2 (6)	C24—C25—H25	120.2
C18—C17—C16	123.8 (5)	C17—C22—C21	116.8 (5)
C22—C17—C16	115.0 (5)	C17—C22—Ir1	114.4 (4)
N1—C5—C4	119.7 (6)	C21—C22—Ir1	128.8 (4)
N1—C5—C6	114.7 (5)	C25—C26—C27	120.4 (6)
C4—C5—C6	125.6 (5)	C25—C26—H26	119.8
N3—C27—C26	119.1 (5)	C27—C26—H26	119.8
N3—C27—C28	116.0 (5)	C18—C19—C20	120.2 (6)
C26—C27—C28	124.9 (6)	C18—C19—H19	119.9
N1—C1—C2	122.1 (6)	C20—C19—H19	119.9
N1—C1—H1	118.9	C9—C8—C7	120.5 (6)
C2—C1—H1	118.9	C9—C8—H8	119.7
N2—C12—C13	122.6 (6)	C7—C8—H8	119.7
N2—C12—H12	118.7	C23—N3—C27	120.2 (5)
C13—C12—H12	118.7	C23—N3—Ir1	126.1 (4)
O1—N4—C28	119.5 (5)	C27—N3—Ir1	113.4 (3)
O1—N4—Ir1	125.8 (4)	C6—C7—C8	119.0 (10)
C28—N4—Ir1	114.6 (4)	C6—C7—H7	120.5
C25—C24—C23	119.0 (6)	C8—C7—H7	120.5
C25—C24—H24	120.5	C20—C21—C22	121.0 (7)
C23—C24—H24	120.5	C20—C21—H21	119.5
C7—C6—C11	121.3 (11)	C22—C21—H21	119.5
C7—C6—C5	122.9 (9)		
			
C22—Ir1—C11—C10	−84.3 (6)	C12—N2—C16—C17	−179.9 (5)
N2—Ir1—C11—C10	−4.2 (6)	Ir1—N2—C16—C17	−0.6 (6)
N1—Ir1—C11—C10	−178.9 (6)	C14—C15—C16—N2	0.0 (8)
N3—Ir1—C11—C10	90.4 (6)	C14—C15—C16—C17	−179.4 (6)
C22—Ir1—C11—C6	96.7 (4)	C18—C17—C16—N2	178.7 (5)
N2—Ir1—C11—C6	176.8 (4)	C22—C17—C16—N2	−1.4 (7)
N1—Ir1—C11—C6	2.1 (4)	C18—C17—C16—C15	−1.9 (9)
N3—Ir1—C11—C6	−88.6 (4)	C22—C17—C16—C15	178.0 (5)
C22—Ir1—N2—C12	−179.1 (5)	C2—C3—C4—C5	2.1 (10)
C11—Ir1—N2—C12	93.6 (5)	N1—C5—C4—C3	−2.9 (10)
N4—Ir1—N2—C12	−79.4 (5)	C6—C5—C4—C3	177.1 (6)
N3—Ir1—N2—C12	−2.3 (5)	O1—N4—C28—C27	−177.4 (5)
C22—Ir1—N2—C16	1.6 (4)	Ir1—N4—C28—C27	−1.1 (7)
C11—Ir1—N2—C16	−85.7 (4)	N3—C27—C28—N4	3.2 (8)
N4—Ir1—N2—C16	101.3 (4)	C26—C27—C28—N4	−175.7 (6)
N3—Ir1—N2—C16	178.4 (4)	C6—C11—C10—C9	0.4 (9)
C16—N2—C12—C13	−1.2 (9)	Ir1—C11—C10—C9	−178.5 (5)
Ir1—N2—C12—C13	179.6 (5)	C11—C10—C9—C8	0.2 (11)
C14—C13—C12—N2	1.1 (10)	C23—C24—C25—C26	−0.8 (12)
C22—Ir1—N4—O1	−10.4 (5)	C18—C17—C22—C21	−0.8 (8)
N2—Ir1—N4—O1	−90.6 (4)	C16—C17—C22—C21	179.3 (5)
N1—Ir1—N4—O1	84.6 (4)	C18—C17—C22—Ir1	−177.3 (4)
N3—Ir1—N4—O1	175.4 (5)	C16—C17—C22—Ir1	2.7 (6)
C22—Ir1—N4—C28	173.6 (4)	C11—Ir1—C22—C17	94.2 (4)
N2—Ir1—N4—C28	93.4 (4)	N2—Ir1—C22—C17	−2.4 (4)
N1—Ir1—N4—C28	−91.4 (4)	N1—Ir1—C22—C17	174.5 (4)
N3—Ir1—N4—C28	−0.6 (4)	N4—Ir1—C22—C17	−90.8 (4)
N3—C23—C24—C25	1.9 (14)	C11—Ir1—C22—C21	−81.8 (5)
C10—C11—C6—C7	−2.0 (8)	N2—Ir1—C22—C21	−178.4 (5)
Ir1—C11—C6—C7	177.2 (4)	N1—Ir1—C22—C21	−1.6 (5)
C10—C11—C6—C5	177.0 (6)	N4—Ir1—C22—C21	93.2 (5)
Ir1—C11—C6—C5	−3.8 (6)	C24—C25—C26—C27	0.0 (11)
N1—C5—C6—C7	−177.2 (5)	N3—C27—C26—C25	−0.4 (9)
C4—C5—C6—C7	2.9 (9)	C28—C27—C26—C25	178.5 (6)
N1—C5—C6—C11	3.8 (7)	C17—C18—C19—C20	0.0 (9)
C4—C5—C6—C11	−176.1 (6)	C21—C20—C19—C18	−0.2 (10)
C16—C15—C14—C13	−0.1 (9)	C10—C9—C8—C7	0.7 (11)
C12—C13—C14—C15	−0.4 (9)	C24—C23—N3—C27	−2.3 (13)
C2—C1—N1—C5	0.3 (9)	C24—C23—N3—Ir1	−175.4 (7)
C2—C1—N1—Ir1	−175.7 (4)	C26—C27—N3—C23	1.5 (9)
C4—C5—N1—C1	1.7 (8)	C28—C27—N3—C23	−177.5 (7)
C6—C5—N1—C1	−178.3 (5)	C26—C27—N3—Ir1	175.4 (4)
C4—C5—N1—Ir1	178.0 (5)	C28—C27—N3—Ir1	−3.5 (6)
C6—C5—N1—Ir1	−2.0 (6)	C11—Ir1—N3—C23	−10.0 (6)
C22—Ir1—N1—C1	88.4 (5)	N2—Ir1—N3—C23	86.6 (6)
C11—Ir1—N1—C1	176.0 (5)	N1—Ir1—N3—C23	−90.5 (6)
N4—Ir1—N1—C1	−11.5 (5)	N4—Ir1—N3—C23	175.8 (6)
N3—Ir1—N1—C1	−88.7 (5)	C11—Ir1—N3—C27	176.5 (4)
C22—Ir1—N1—C5	−87.7 (4)	N2—Ir1—N3—C27	−86.9 (4)
C11—Ir1—N1—C5	−0.1 (4)	N1—Ir1—N3—C27	96.0 (4)
N4—Ir1—N1—C5	172.4 (4)	N4—Ir1—N3—C27	2.3 (3)
N3—Ir1—N1—C5	95.1 (4)	C11—C6—C7—C8	2.9 (8)
C4—C3—C2—C1	−0.2 (10)	C5—C6—C7—C8	−176.1 (6)
N1—C1—C2—C3	−1.1 (9)	C9—C8—C7—C6	−2.2 (10)
C22—C17—C18—C19	0.5 (9)	C19—C20—C21—C22	−0.1 (10)
C16—C17—C18—C19	−179.6 (6)	C17—C22—C21—C20	0.6 (8)
C12—N2—C16—C15	0.6 (7)	Ir1—C22—C21—C20	176.5 (5)
Ir1—N2—C16—C15	179.9 (4)		

### Hydrogen-bond geometry (Å, º)

Cg1, Cg2 and Cg3 are the centroids of the C17–C22, N2/C12–C16 and N1/C1–C5 rings, respectively.

**Table:**

*D*—H···*A*	*D*—H	H···*A*	*D*···*A*	*D*—H···*A*
C7—H7···O1^i^	0.93	2.35	3.235 (10)	159
C15—H15···O1^ii^	0.93	2.37	3.239 (9)	155
C3—H3···*Cg*1^iii^	0.93	2.74	3.604 (7)	155
C8—H8···*Cg*2^i^	0.93	2.69	3.606 (7)	170
C13—H13···*Cg*3^iv^	0.93	2.71	3.538 (9)	148

Symmetry codes: (i) *x*, *y*, *z*+1; (ii) −*x*+1, *y*−1/2, −*z*; (iii) −*x*+1, *y*+1/2, −*z*+1; (iv) −*x*, *y*−1/2, −*z*.
